# The willingness to continue using wearable devices among the elderly: SEM and FsQCA analysis

**DOI:** 10.1186/s12911-023-02336-8

**Published:** 2023-10-16

**Authors:** Ying Wang, Liyan Lu, Rui Zhang, Yiming Ma, Shuping Zhao, Changyong Liang

**Affiliations:** 1 Anhui University of Chinese Medicine, Hefei, 230012 China; 2 Hefei University of Technology, Hefei, 230009 China

**Keywords:** Elderly, Wearble devices, Technical characteristic, Personal characteristic, Health promotion, FsQCA

## Abstract

**Background:**

With population aging and the scarcity of resources for elderly individuals, wearable devices pose opportunities and challenges for elderly care institutions. However, few studies have examined the effects of technical characteristics, personal characteristics, and health promotion on the willingness of elderly individuals to continue using wearable devices.

**Objective:**

This study explored the effects of technical characteristics and personal characteristics on the willingness of elderly individuals to continue using wearable devices through health promotion, drawing on the technology acceptance model and the value attitude behaviour model.

**Methods:**

We obtained 265 valid samples through questionnaire surveys and used structural equation modelling (SEM) and fuzzy set qualitative comparative analysis (FsQCA) to clarify the complex causal patterns of elderly people’s willingness to continue using wearable devices.

**Results:**

The SEM results showed that perceived usefulness, perceived reliability, self-perceived ageing, and health promotion affected willingness to continue using wearable devices. However, perceived ease of use had no effect. FsQCA showed that elderly individuals are highly willing to continue using wearable devices, yielding five solutions. Perceived ageing was essential in four of these solutions. The impact of perceived ease of use on continued use intention was dynamic and complex.

**Conclusions:**

This study used two methods to provide insight into the willingness of elderly individuals to continue using wearable devices. In addition, this study discussed associated implications, limitations, and future research directions.

## Introduction

In recent years, rapid population aging has occurred in China, and to cope with the associated challenges, 329,000 elderly care institutions and facilities have been constructed across China [1]. However, at present, elderly care institutions have a shortage of nursing resources and are unable to provide comprehensive nursing services, which seriously affects the long-term development of elderly care institutions.

With continuous advances in communication and information technology, wearable devices have been developed, posing opportunities and challenges for elderly care institutions. The devices use sensor technology to collect user physiological, psychological and behavioural parameters and inform health management through data analysis. They can provide sleep, step count, and blood sugar monitoring and GPS positioning functions and not only assist health monitoring but also allow individuals to keep in touch with family or friends (e.g., through smart watches and other devices) [2]. As a technological supplement to elderly care resources, wearable devices have great potential to solve issues arising from population aging. The global wearable device market is expected to generate $2.2 billion in revenue, while the number of wearable devices is expected to exceed 1 billion [3, 4]. In the Chinese market, 119 million older people are using mobile devices to access the internet, and this number is growing, especially during the COVID-19 pandemic. The need to provide monitoring and assistive technologies for older people is also urgent, which further illustrates the enormous potential and market prospects of wearable devices in the elderly population [5]. On the one hand, these devices can help manage the health of older people, for example, by continuously monitoring physical and psychological parameters over time and alerting caregivers when danger arises to prevent further harm [6]. On the other hand, wearable devices can also assist or support nursing staff in monitoring the health status of elderly individuals in a variety of dimensions, such as sleep, falls, blood pressure fluctuations and heart rate (e.g., detection of abnormalities), to achieve remote control and management. There is plenty of evidence that using wearable technology can help elderly care institutions manage the health and well-being of older adults. Hence, elderly care institutions have increasingly introduced wearable devices to improve the quality and efficiency of elderly care services.

While digital health products are gaining popularity and acceptance among elderly individuals, the current usage of wearable devices in care facilities remains low. This can result in reduced performance and increased workload for elderly care institutions. Therefore, maintaining use of such devices among elderly users is an important challenge faced by these institutions. Understanding the factors that influence older adults’ continued use of wearable devices is crucial for practitioners and researchers to comprehend how older adults make decisions regarding the continuation of such technologies.

There are still gaps in the literature regarding the mechanisms that influence the continuous use of wearable devices by elderly individuals. Many studies have explored the willingness of elderly individuals to adopt or accept wearable devices (such as ease of use, usefulness, reliability, product security and privacy) from the perspective of technology perception [6, 7]. However, these studies have neglected to examine the use of elderly individuals after the adoption stage. In addition, the use of technology by elderly individuals has unique characteristics, especially given the perception that ageing will affect their behaviour [8]. However, few scholars have evaluated perceived ageing as an individual characteristic in elderly individuals to explore its influence on their continuous use of wearable devices. Therefore, studying the constant use of wearable devices by older people from the perspective of technical characteristics and individual perceptions is needed to fill gaps in the literature. In addition, assessing the effects of perceived ageing could provide some insight into the continued use of wearables by elderly individuals.

Health promotion is a concept involving attributing value to one’s own health and is an attitude that can help older people better manage their physical health [9]. It is important to improve lifestyle factors through the use of wearable devices and to motivate older people to engage in management of their own. However, no researchers have incorporated health promotion into research models when exploring the factors influencing the willingness of elderly individuals to continue using wearable devices. Hence, understanding the relationship between health promotion and the willingness of older people to continue using wearable devices is one of the aims of the present research.

To address the above research gap, this study examined elderly people’s willingness to continue using wearable devices from the perspective of technical characteristics and individual characteristics. Based on the above arguments, we formulated the following research questions:

RQ1: How do technical and personal characteristics influence willingness to continue using wearable devices through health promotion?

RQ2: How do technical and personal characteristics influence health promotion?

This study contributes to research on wearable devices. First, this study expands knowledge of continued use intention by examining the factors influencing this intention (technical characteristics and personal characteristics) in elderly individuals. Second, this study enhances understanding of the TAM and VAB model and investigated how technology characteristics and individual characteristics affect continued use intention through health promotion in an empirical study, which can inform studies of individual use behaviour. Finally, the PLS-SEM and FsQCA approaches used can yield not only better understanding of the relationship between variables to better predict continued use intention but also provide insight into the influence of different combinations of factors on continued use intention in older people to better understand the mechanism of continued use intention for wearable devices. Additionally, few studies have used FsQCA to explore the willingness of older individuals to continue using wearable devices; the present study helps to fill this gap.

## Background theory and literature review

Through a literature review, we discovered that some scholars have utilized theories such as commitment-trust theory and expectation confirmation theory to investigate individuals’ continued use intention [10]. However, these studies have failed to address elderly people’s continued intention to use wearables in the health care field. In contrast, the technology acceptance model (TAM) and value attitude behaviour (VAB) model have been employed to explore the usage of wearable devices by elderly individuals [6, 8]. However, the research on postadoption behaviour is still insufficient. Therefore, the TAM and VAB model were used in this study. Additionally, ageing is a prominent feature among elderly individuals, and their continued use of wearable devices may be influenced by perceived ageing. Elderly people’s perception of ageing may also increase health promotion. Nevertheless, most scholars have studied effects on continued use intention from a technical aspect. In contrast, few scholars have examined the impact of perceived ageing and health promotion on older people’s continued use of wearable devices. Therefore, this paper incorporated perceived ageing and health promotion to develop a comprehensive model based on the TAM and VAB model to better comprehend continued use intention for wearable devices in elderly individuals and factors that influence their use.

### The technology acceptance model

The initial purpose of the technology acceptance model (TAM) was to predict users’ acceptance of computers. It is one of the most common theories for studying user behaviour [11]. The TAM describes the relationship between perceived usefulness, perceived ease of use, and behavioural intention. Perceived usefulness is defined as the degree to which a person believes that using the system (e.g., a wearable device) will improve their (work) performance, and perceived ease of use is defined as the degree of effort a person believes will be required to use the system [12]. Perceived usefulness and perceived ease of use are important constructs in the TAM and are widely used to predict technological attitudes. However, some scholars have proposed that other variables need to be added to the model, such as perceived reliability, which refers to the user’s evaluation of technical security and privacy when using information technology or systems. Previous research has shown that users care about the privacy, comfort, safety, and performance of wearable devices [13]. Some users indicated that they would avoid using the technology if they perceive a privacy threat [14]. In addition, wearable medical devices that contact the skin might irritate the user’s skin, which is a potential risk, especially for elderly individuals. Therefore, this paper incorporated perceived reliability into the model. The TAM can facilitate understanding of how individual perceptions of technology impact behaviour, and different scholars have integrated various technologies into relevant contextual frameworks [15]. Currently, most scholars have employed the TAM to investigate elderly people’s willingness to use wearable devices, but a few scholars have applied it to study the willingness of elderly people to continue to use these devices in the postadoption stage. Therefore, further evaluation is necessary to determine whether the TAM can be effectively utilized in investigations of the intention to continue using wearable devices.

### The value attitude behaviour model

The VAB model can also be used to predict user behaviour. The model includes three main variables: perceived value, attitude, and behaviour [16]. Perceived value affects user behaviour [17]. High value is the main motivation for user behaviour. Perceived value involves a cognitive evaluation of the product by the user and mainly refers to the value or utility of the product to the user when performing a user behaviour [18]. This study focused on perceived usefulness, perceived ease of use, and perceived reliability to assess the perceived value of wearable devices for elderly individuals. If elderly users perceive wearable devices to have higher value, they are more likely to have positive attitudes and behaviours towards them. The value attitude behaviour (VAB) model suggests that an object’s perceived value influences individuals’ attitudes and behaviours. Therefore, this paper hypothesized that the perceived technology characteristics of wearable devices by elderly individuals will predict their willingness to continue using them.

Health promotion is described by Vance, an American health educator, as a lifestyle, attitude, or concept. In this paper, health promotion is defined as the perspective of older people that wearable devices can promote a healthy lifestyle or their tendency to engage in health-promoting behaviours. Attitude is an important predictor of behaviour, and perceived value is a form of cognition that influences the evaluation of related objects and affects individual attitude [19, 20]. Therefore, the inclusion of health promotion in the VAB framework in this paper helped to expand the model and paves the way for its contributions in the field. It is worth noting that few scholars have incorporated the concept of health promotion into the VAB model.

### Research hypothesis

**Fig. 1:**
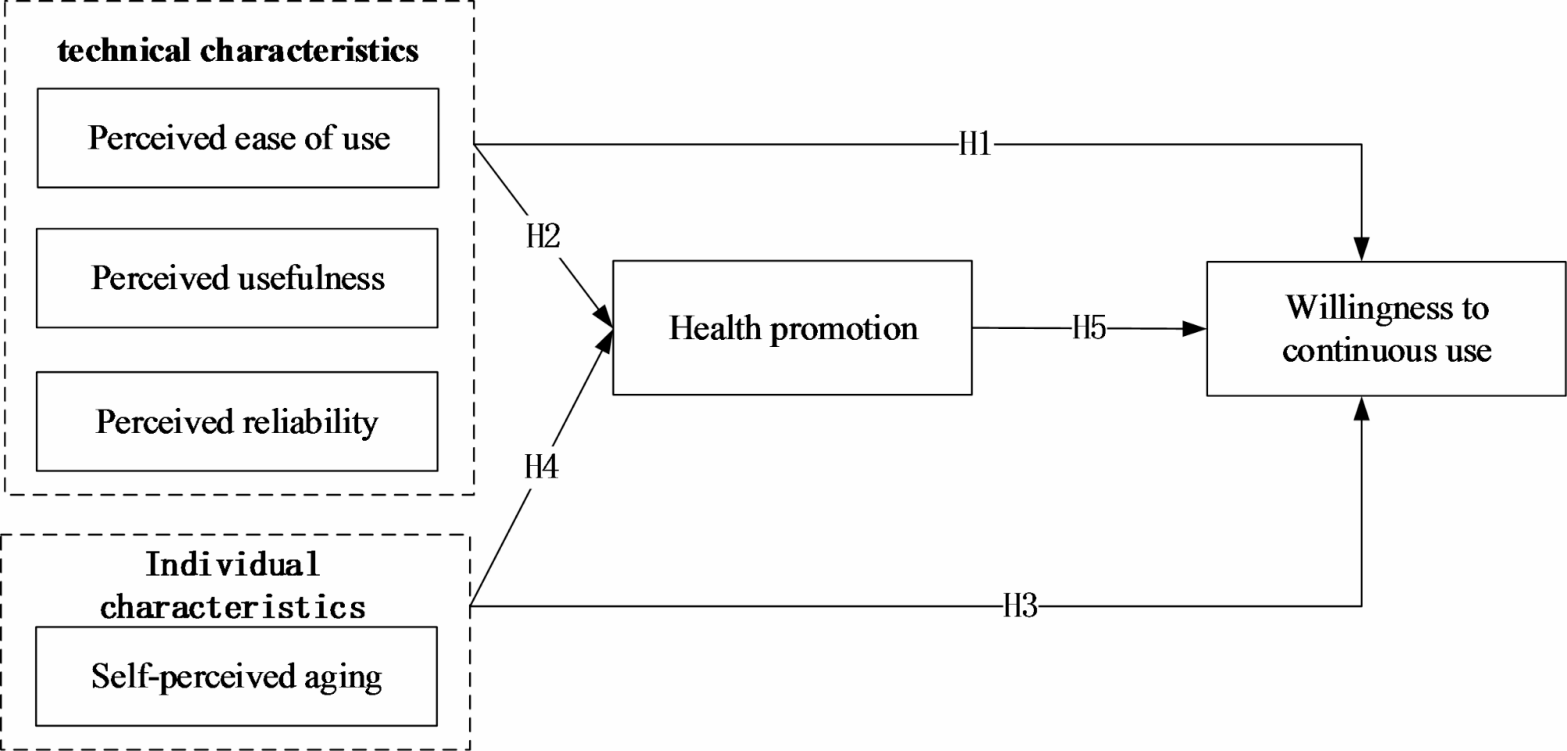
The research model

This paper proposed a research model based on the theory of technology acceptance model (TAM) and value attitude behaviour (VAB) theory(Fig. 1). The model examined the effects of technical characteristics (perceived usefulness, perceived ease of use, and perceived reliability) and individual characteristics (self-perceived ageing) on health promotion. The dependent variable was the willingness of elderly individuals to continue using wearable devices, which was predicted to be influenced by both technical characteristics and individual characteristics. Health promotion may be caused by these factors, and an in-depth study of their mechanisms of action could shed light on the continued use of wearable devices by elderly individuals.

### Product usage perception and continued use intention

According to the TAM, the individual’s perception of the ease of use and usefulness of the system or product will have a substantial impact on user satisfaction and behavioural intentions [21]. Continuance intention (CI) is the user’s willingness to continue using the system or use it frequently in the future after using the system for the first time [22].

In the TAM, perceived ease of use and perceived usefulness are widely used to study the behavioural intentions of users of technology [23, 24]. The VAB model proposes that perceived value affects individual attitudes and behaviours. Cerulli-Harms et al. proposed that the reliability of technology can attract users and that users may have a preference for reliable technology [25]; thus, if elderly users believe that wearable devices are more reliable and safer to use, they are more likely to continue using them. Therefore, perceived reliability can also be considered a possible factor affecting the continued use intention of elderly individuals.

In elderly care institutions, elderly people’s perception of the use of wearable devices may affect their inherent willingness to use those devices. Based on the above content, the following hypotheses were proposed:

H1a: Perceived ease of use has a significant positive impact on the willingness to continue using wearable devices.

H1b: Perceived usefulness has a significant positive impact on the willingness to continue using wearable devices.

H1c: Perceived reliability has a significant positive impact on the willingness to continue using wearable devices.

### Product usage perception and health promotion

The usability and comfort of wearable technology can encourage users to increase their daily physical activity [26]. In the current elderly care environment, health management is very important. Elderly individuals believe that the availability of activity monitoring technology can support their health management [27]. In the pandemic, elderly individuals experienced social isolation and loneliness. The use of assistive technology can alleviate loneliness and enable elderly individuals to maintain social and emotional connections [28]. “Fall alarm” technology can detect elderly individuals’ risk of falling before a fall occurs, allowing elderly individuals to improve their control ability [29]. Elderly people’s perception of the use of products will encourage them to participate in healthy behaviours that are conducive to improving their own health. Similarly, the reliability of the product itself, especially the safety of health products, is also a factor driving users’ health promotion behaviour. Therefore, the perception of the use of wearable devices by elderly individuals in elderly care institutions may affect their intentions to improve their lifestyle in a favourable way. Based on the above content, the following hypotheses were proposed:

H2a: Perceived ease of use has a significant positive impact on health promotion.

H2b: Perceived usefulness has a significant positive impact on health promotion.

H2c: Perceived reliability has a significant positive impact on health promotion.

### Self-perceived ageing and health promotion and willingness to continue using wearable devices

Self-perceived ageing refers to the subjective perception and emotions of elderly individuals in response to physical, psychological, and social ageing. This perception and response will affect the behavioural tendencies of elderly individuals in the ageing process [30]. Studies have found that elderly people living alone, especially those with dementia, have higher requirements for safety and social contact. To ensure safety in daily life, they rely on assistive technology for a long time [31]. Older stroke patients who feel supported by technology will be willing to continue using the technology [32].

For elderly individuals, positive self-perception of ageing is beneficial to physical and mental health. Studies have shown that positive self-perception of ageing is associated with positive health outcomes [33]. When elderly individuals have more positive perceptions of aging, they will be more effective in improving their physical and mental health [34]. In summary, this article hypothesized the following:

H3: self-perceived ageing has a significant positive impact on the willingness to continue using wearable devices.

H4: self-perceived ageing has a significant positive impact on health promotion.

### Health promotion and the willingness to continue using wearable devices

Speier et al. concluded in a patient-based electronic health study that wearable trackers can improve patients’ health attitudes and found high compliance during the three-month tracking process [35]. Studies have shown that when elderly individuals use information technology, they can improve their daily activities and maintain independence, making them more confident in technology, which also increases their willingness to continue using technology and equipment in the future [36]. In the daily life of elderly individuals, health is one of the important goals pursued. Therefore, this article hypothesized the following:

H5: Health promotion has a significant positive impact on the willingness to continue using wearable devices.

## Methods

### Measures

All variables were measured using previously developed scales, with the language slightly modified for the elderly care environment. The perceived ease of use scale (3 items) was adapted from [37]. The perceived usefulness scale (4 items) was from [38] and [37]. The perceived reliability scale (3 items) was from [37, 39]. The self-perceived ageing scale (4 items) was adapted from the simplified perceived ageing scale [40]. The health promotion scale (5 items) was adapted from the Adolescent Health Promotion Scale [41]. The last scale, measuring willingness to use (5 items), was from [42] and [8]. The questionnaire items were rated a seven-level Likert scale, from 1 = strong disapproval to 7 = strong approval.

### Data collection

The target population of this study was elderly individuals residing in elderly care institutions in Anhui Province, China. The researcher first obtained permission from the administrator of the institution to collect data from the elderly residents. After obtaining a list of names of the elderly residents, the researcher contacted each resident and explained the purpose of the study. Participants signed an informed consent form and completed a questionnaire, which was completed in approximately 15–20 min. Each participant was provided with $5 of daily necessities as compensation for their time. A total of 338 respondents were included in the study, with 265 valid questionnaires received, for a response rate of 78.4%.

### Data analysis

In this study, partial least squares SEM (PLS-SEM) and FsQCA were used to test the proposed model. First, we used SmartPLS 3.0 to examine the impact of product usage perception, individual characteristics, and health promotion on the willingness to continue using wearable devices, which yielded the measurement model, structural model, and model fit [43]. PLS-SEM has the advantages of flexible handling of missing data and relatively loose requirements for normally distributed data and can also estimate complex models with small samples [44, 45]. Second, FsQCA was used to understand the full combination of variables that predicted high willingness to continue using wearable devices. Unlike traditional regression and SEM methods, FsQCA is an asymmetric method that can identify multiple path combinations that lead to an outcome. Using FsQCA can capture the complexity of elderly people’s continued use of wearable devices.

## Results

Table 1 presents the demographic characteristics of 265 elderly participants in this study. Among the participants, 50.21% were male, while 49.79% were female; 66.32% were between the ages of 61 and 70 years. Regarding education levels, approximately 85.48% of the participants had completed less than senior high school. Additionally, 52.28% of the participants were single, and 28.63% had a monthly income of more than 4501 RMB.

**Table 1:** Demographic characteristics of respondents (total N = 265)

Variable	Level	Number	Percentage
Sex	Male	121	50.21%
	Female	120	49.79%
Age	55–60 years	17	7.05%
	61–65 years	58	24.07%
	66–70 years	97	40.25%
	Over 71 years	69	28.63%
Education	Primary school	128	53.11%
	Junior high school	78	32.37%
	Senior high school	27	11.20%
	Technical secondary school	8	0.03%
Marital status	Married	115	47.72%
	Divorced	56	23.24%
	Separation	47	19.50%
	Widowed	23	9.54%
Monthly income	Below 2500 RMB	33	13.69%
	2501–3500 RMB	80	33.20%
	3501–4500 RMB	59	24.48%
	4501–5500 RMB	39	16.18%
	5000 RMB or more	30	12.45%

### PLS-SEM results

#### Evaluation of measurement models

**Descriptive statistics.** As shown in Table 2, the average score on each item value of each variable item of the model ranged from 4.947 to 5.313, and the standard deviation ranged from 1.256 to 1.596, indicating that the data were relatively concentrated, with small fluctuations, and had good adaptability. The factor loading of each item ranged from 0.724 to 0.922, and all loadings were higher than 0.7, indicating that there was a strong correlation between the observed variable and the structural variable to which it belonged [46]; therefore, the items were reasonable.

**Table 2:** The reliability and validity results

Index	Mean	SD	Loading (>0.7)	VIF (<5.0)
PEU1	5.313	1.426	0.838	1.338
PEU2	5.060	1.447	0.847	1.697
PEU3	5.226	1.332	0.724	1.505
PU1	5.257	1.444	0.904	3.490
PU2	5.215	1.574	0.922	4.066
PU3	5.204	1.584	0.868	2.501
PU4	4.947	1.450	0.817	1.963
PR1	4.989	1.518	0.888	2.503
PR2	5.026	1.521	0.933	3.548
PR3	5.034	1.596	0.914	2.793
SPA1	5.162	1.315	0.822	1.886
SPA2	5.068	1.429	0.859	2.116
SPA3	5.204	1.476	0.830	2.040
SPA4	5.140	1.340	0.780	1.731
HP1	5.158	1.373	0.836	2.220
HP2	5.189	1.281	0.786	2.037
HP3	5.219	1.279	0.801	2.006
HP4	5.091	1.376	0.847	2.418
HP5	5.208	1.311	0.757	1.756
WTC1	5.132	1.38	0.781	2.106
WTC2	5.030	1.256	0.812	2.316
WTC3	5.113	1.327	0.858	2.697
WTC4	5.125	1.486	0.836	2.552
WTC5	5.087	1.410	0.856	2.341

**Reliability and validity.** To test the reliability of the scales, Cronbach’s alpha values were used. From the results, all Cronbach’s alpha values ranged from 0.729 to 0.901, which is greater than the recommended value of 0.7 [47]. This means that the reliability of the scales was satisfactory.

To test the validity of the scales, aggregate validity was first evaluated. Factor loading, composite reliability (CR), and the average variance extracted (AVE) are three effective indicators for testing aggregate validity [48]. As shown in Table 3, the factor loading values exceeded the recommended value of 0.6 [49].

To determine discriminant validity, we used the standard of Fornell and Larcker: the square root of the AVE of each observed variable was greater than the correlation coefficient between it and other observed variables, which showed that the discriminant validity of each observed variable was relatively high [50]. See Tables 3 and 4 for details.

**Table 3:** Reliability and validity results

Variable	Cronbach’s alpha (> 0.70)	Composite reliability (CR) (> 0.70)	Average variance extracted (AVE) (> 0.50)
Health promotion	0.865	0.903	0.65
Perceived reliability	0.898	0.937	0.831
Perceived ease of use	0.729	0.846	0.648
Perceived usefulness	0.901	0.931	0.772
Continued use intention	0.886	0.916	0.687
Perceived ageing	0.841	0.894	0.678

**Table 4:** Discriminant validity of the measurement model (Fornell-Larcker criterion)

Construct	HP	PR	PEU	PU	WTC	SPA
Health promotion (HP)	**0.806**					
Perceived reliability (PR)	0.529	**0.912**				
Perceived ease of us (PEU)	0.531	0.523	**0.805**			
Perceived usefulness (PU)	0.671	0.523	0.476	**0.879**		
Continued use intention (WTC)	0.655	0.571	0.455	0.588	**0.829**	
Perceived ageing (SPA)	0.678	0.603	0.51	0.565	0.627	

### Structural equation modelling

The path coefficients of the PLS-SEM may be biased due to multicollinearity in the structural model. To address this issue, we checked for multicollinearity by evaluating the variance inflation factor (VIF) for each measurement item. The VIF values should not exceed 5 [51], and the results showed that all VIF values in the model were lower than this threshold, indicating the absence of multicollinearity.

**Fig. 2:**
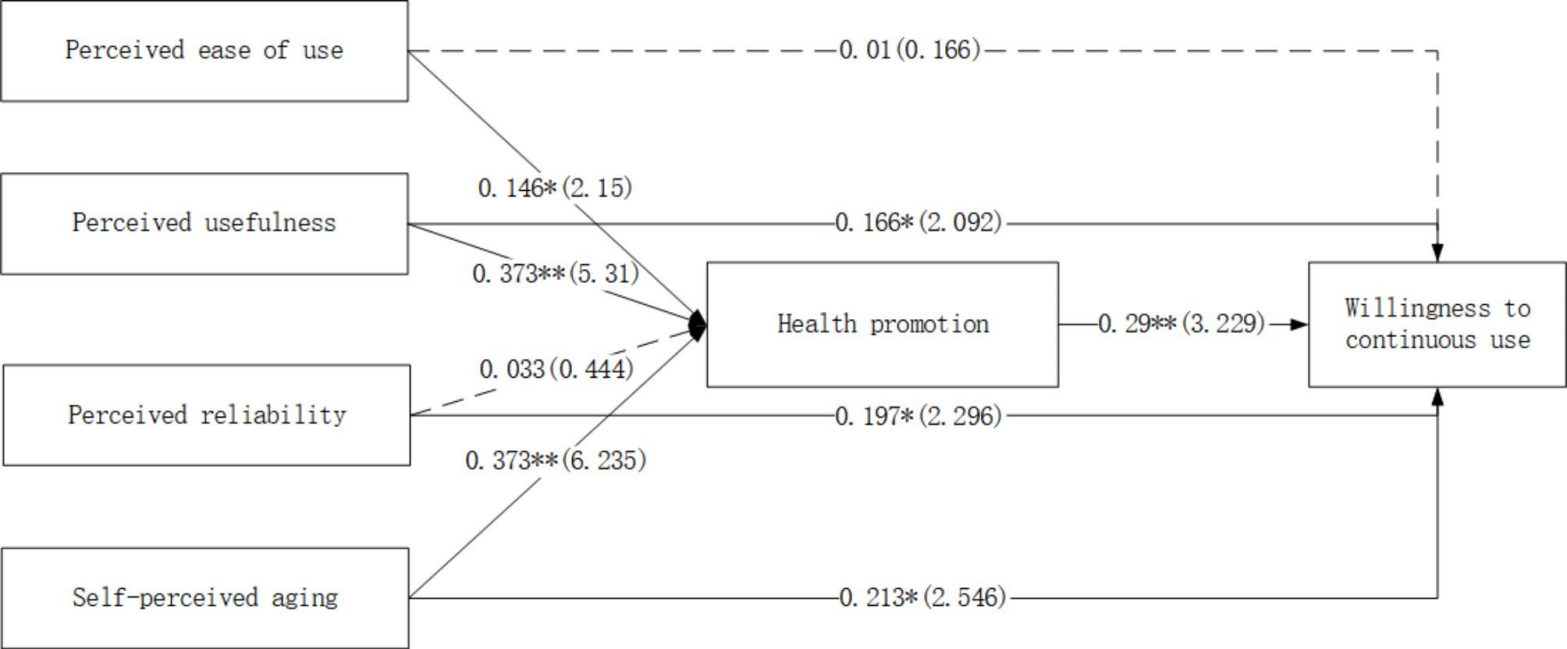
Structural equation modelling results

To examine the path coefficients, their significance levels, and t values, we used a bootstrapping method with 5000 subsamples. The results showed that all paths except for perceived ease of use significantly predicted continued use intention (H1a) and all paths except for perceived reliability significantly predicted health promotion (H2c). See Fig. 2 for more details. Table 4 presents the final hypothesis test results.

**Table 5:** Structural parameter estimation results

Hypothetical path	Path coefficient	T value	P value	Supported
H1a: Perceived ease of use → Continued use intention	0.010	0.166	0.868	No
H1b: Perceived usefulness → Continued use intention	0.166	2.092	0.037	Yes
H1c: Perceived reliability → Continued use intention	0.197	2.296	0.022	Yes
H2a: Perceived ease of use → Health promotion	0.146	2.150	0.032	Yes
H2b: Perceived usefulness → Health promotion	0.373	5.310	0.000	Yes
H2c: Perceived reliability → Health promotion	0.033	0.444	0.657	No
H3: Perceived ageing → Continued use intention	0.213	2.546	0.011	Yes
H4: Perceived ageing → Health promotion	0.373	6.235	0.000	Yes
H5: Health promotion → Continued use intention	0.290	3.229	0.001	Yes

Note: * means p < 0.05, ** means p < 0.01

In addition, structural equation modelling also yielded the coefficient of determination (R^2^), which measures the variance explained. This study had two endogenous latent variables: health promotion and willingness to continue. The survey results shows that the R^2^ values of these two dimensions were 0.592 and 0.529.

### Model Fit

The goodness-of-fit (GoF) index was used to evaluate the fit of the model. The standardized root mean square residual (SRMR = 0.063) was lower than 0.08, indicating a good fit [52]. GoF values reflect the fit of a model: values less than 0.10 are considered low, values between 0.10 and 0.25 are small, values between 0.25 and 0.36 are medium, and values greater than 0.36 are high [53, 54]. According to the formula below, the model had good fit (0.631, a high value). Therefore, the research model exhibited good fit [55].

$$GoF = \sqrt {\overline {{R^2}} \times \overline {AVE} } = \sqrt {0.5605 \times 0.711} = 0.631$$

### FsQCA analysis

Based on the results of PLS-SEM, FsQCA was used to examine the effect of antecedent conditions on the willingness to continue using wearable devices to obtain a more comprehensive description of antecedent variables and results. FsQCA is used to explore the interactions between variables. These interactions are meaningful for the study of the willingness to continue using wearable devices.

### Data calibration

In this study, data were collected on a 7-point Likert scale, but the range of values input into FsQCA are between 0 and 1. Therefore, before qualitative comparison and analysis, the data were converted to values on a 0–1 scale [56]. First, we calculated the mean value of each measurement item as the reflected value of the variable. Then, according to the 5% (fully out), 95% (fully in), and 50% (crossover point) standards proposed by Ragin, we used the calibrate function in FsQCA software to calibrate the data [57]. After calibration, we explored the paths that affected the willingness of elderly individuals to continue using wearable devices.

### Necessity analysis

A necessary condition is a condition that must exist for the result to occur, but its existence does not necessarily cause the result to occur. Usually, a consistency score of antecedent conditional variables greater than 0.9 is considered a necessary condition for the result [56]. Table 5 shows that the highest agreement among all conditions was 0.857330, so none of the individual conditions were necessary to generate the willingness to continue using wearable devices. To this end, we performed configuration analysis on the various paths that generated the willingness to continue using wearable devices.

**Table 6:** Analysis of necessary conditions

Condition variable	WTC		~WTC	
	Consistency	Coverage	Consistency	Coverage
PEU	0.771612	0.762199	0.675201	0.616963
~PEU	0.612233	0.670806	0.739752	0.749761
PU	0.827764	0.798697	0.612062	0.546296
~PU	0.529784	0.596174	0.774463	0.806180
PR	0.846796	0.791540	0.632951	0.547294
~PR	0.515691	0.602990	0.758914	0.820861
SPA	0.857330	0.812922	0.617167	0.541328
~SPA	0.516272	0.593140	0.786713	0.836088
HP	0.832704	0.833068	0.593215	0.548983
~HP	0.549179	0.593406	0.819617	0.819231

Notes: PEU denotes perceived ease of use, PU denotes perceived usefulness, PR denotes perceived reliability, SPA denotes perceived ageing, HP denotes health promotion, WTC denotes continued use intention.“~”indicates the negation of the condition

### Conditional configuration analysis

In the subsequent stage of the study, we constructed and analysed the truth table. This requires setting thresholds for sample case data. It usually includes two aspects: the frequency threshold and the consistency threshold of the sample. Using FsQCA3.0, following Ragin’s recommendation, we set the consistency to 0.8 and the frequency to 1 [56]. FsQCA software was used to simplify the data in the truth table by Boolean algebra minimization, and complex solutions, intermediate solutions, and general solutions can be obtained; thus, five paths of continued use intention were obtained.

Table 6 summarizes the results of the intermediate solutions for high willingness of elderly individuals to continue using wearable devices. The solution consistency was 0.890815, and the solution coverage was 0.798925, which means that all combinations of antecedent variables can explain 79.89% of the variance in questionnaire responses. Specifically, in the five preceding dependent variable configurations, the consistency scores were all greater than 0.9, and the value of the unique coverage rate was nonzero, indicating that configurations H1 through H5 had strong explanatory power.

**Table 7:** Conditions and configurations of factors affecting the willingness to continue using wearable devices

Variable	Solution				
	1	2	3	4	5
PEU	ⓧ			ⓧ	●
PU	ⓧ	●		•	●
PR			●	●	●
SPA	●	●	●	●	
HP	•	●	●		•
Raw coverage	0.504794	0.707904	0.706451	0.481185	0.618698
Unique coverage	0.0128578	0.0233185	0.0185967	0.0215747	0.0201946
Consistency	0.937787	0.914938	0.91409	0.965738	0.941313
Solution coverage	0.798925				
Solution consistency	0.890815				

Notes: ● indicates the inclusion of a factor as a core factor in this structure; • indicates the inclusion of a factor as an auxiliary factor in this structure (edge conditions exist); ⓧ indicates that the core factor is not included in this structure (the core condition is absent); ⓧ indicates that auxiliary factors are not included in this structure (the marginal condition is absent); blanks indicate that the factors may or may not exist

Table 7 shows that under the interactive matching of product usage perception, individual characteristics, and health promotion, five solutions or paths of continued use intention were generated; the consistency of the five paths was greater than 0.9, which is above the threshold of 0.8, and the solution consistency was 0.890815, indicating that the configuration of these paths met the specification requirements [58].

Analysis of each configuration horizontally revealed the following. **Path 1** showed that when the product’s ease of use is low, perceived ageing and health promotion cause elderly individuals to have a high willingness to continue using it. **Path 2** showed that regardless of the perceived ease of use and perceived reliability of devices, high perceived usefulness, perceived ageing, and health promotion increase the willingness of elderly individuals to continue using devices. **Path 3** showed that regardless of whether perceived ease of use and perceived usefulness, high levels of perceived reliability, perceived ageing, and health promotion can produce a higher willingness to continue using wearable devices. **Path 4** showed that regardless of health promotion, high perceived reliability and perceived ageing-assisted perceived usefulness led to a higher willingness to continue using wearable devices. **Path 5** showed that regardless of perceived ageing, wearable devices with a high level of perceived ease of use, perceived usefulness, and perceived reliability that increase health promotion can lead to high levels of willingness to use them among elderly individuals.

In summary, the FsQCA results confirmed the following points. First, this study identified five causal configurations that predicted a high willingness to continue using wearable devices. Second, the presence or absence of product use perception, individual characteristics, and health promotion can lead to a high willingness to continue using wearable devices, depending on the different combinations of these conditions and other conditions. The results shows that cause and effect are asymmetric. Third, individual characteristics and perceived ageing were included in four of the configurations.

### Comparison of PLS-SEM and FsQCA results

The PLS-SEM and FsQCA results in this paper were similar. PLS-SEM showed that the effect of perceived ease of use on continued use intention was not significant (H1a). In the FsQCA results, Paths 1 and 4 did not include the core variable of perceived ease of use, but the combination of other conditions produced a high continued use intention.

However, some PLS-SEM results contradicted the FsQCA results. For example, PLS-SEM showed a single plan to increase the willingness to continue using wearable devices, whereas FsQCA revealed five paths or solutions. These findings are in line with Larkin’s view that different configurations with different prerequisites can achieve the same results. Following the principle of causal asymmetry, FsQCA suggested that perceived ease of use, perceived usefulness, perceived reliability, perceived ageing, and health promotion will have different effects on continued use intention in different solutions. As shown in Table 6, Paths 2 and 3 showed that regardless of the presence or absence of perceived ease of use, perceived usefulness, perceived reliability, perceived ageing, and health promotion will produce a high willingness to continue using wearable devices. Path 5 shows that the presence of perceived ease of use combined with perceived usefulness, perceived reliability, and health promotion will also produce a high willingness to continue using wearable devices. In summary, perceived ease of use is complicated. Although PLS-SEM can verify the causal relationship between antecedents and the willingness to continue using wearable devices, it cannot confirm such insights.

## Discussion

The purpose of this study was to use the PLS-SEM and FsQCA methods to provide a comprehensive assessment of the continued use of wearable devices. We examined the symmetry of relationships between product usage perception (i.e., perceived ease of use, perceived usefulness, and perceived reliability), individual characteristics (i.e., perceived ageing), health promotion, and willingness to continue using wearable devices. This research yielded several findings.

First, PLS-SEM showed that the influence of perceived ease of use on the willingness of elderly individuals to continue using wearable devices was not significant in the elderly care institution environment (H1a). This aligns with Li et al.’s research on wearable technology for elderly individuals [6]. However, it is in contrast to the view that ease of use is an important factor affecting user behavioural intentions, as proposed by Bawack and Kala Kamdjou [59]. Our results showed that the impact of perceived ease of use on behavioural intentions is complex. The FsQCA results in this study showed that regardless of perceived ease of use, high willingness to continue using wearable devices can be achieved, depending on the combination of other causal conditions. For example, Paths 1 and 4 suggested that the core conditions lack perceived ease of use, but the configuration of other variables affects the willingness to continue using wearable devices. However, in Path 5, perceived ease of use was a core variable combined with perceived usefulness and perceived reliability, increasing health promotion and thereby the willingness to continue using wearable devices; that is, in this path, perceived ease of use was a necessary condition for the willingness to continue using wearable devices. Thus, FsQCA provided evidence of a nonlinear relationship between perceived ease of use and the willingness to continue using wearable devices.

Second, in contrast to previous studies, the PLS-SEM results in this paper showed that perceived reliability did not affect health promotion (H2c). For example, Jakicic et al. showed that the use of wearable devices can encourage users to actively exercise and increase health-promoting behaviours [60]. The reasons for the inconsistent conclusions in this article are as follows. (1) Although many factors affect individual health promotion behaviours, most people have higher expectations of usefulness, and usefulness is an important factor in health promotion behaviours [61]. In this study, although the use of wearable devices promoted healthy behaviours in elderly individuals, elderly individuals were more concerned about the usefulness of the product and less concerned about the reliability of the product. Therefore, the impact of perceived reliability on the health promotion of elderly individuals was not significant. (2) The elderly population is heterogeneous. Some scholars have pointed out that elderly individuals do not pay attention to privacy and security issues when using information technology [62]. Scholars have reported that most elderly people do not care about the reliability of wearable systems [6]. In elderly care institutions, as the cognitive function of elderly individuals deteriorates, they pay more attention to changes in their physical health. When faced with threats to their health, even if there are problems with product reliability (such as security failures and privacy leaks), elderly individuals will not decrease their level of use.

Third, the horizontal analysis in FsQCA concluded that perceived ageing is the most important antecedent condition of elderly people’s willingness to continue using wearable devices, followed by perceived reliability. This shows that when elderly individuals face health threats caused by ageing, they tend to continue using smart, wearable devices to monitor their health indicators and maintain their daily life. Levy et al. believe that perceived ageing affects the coping strategies of elderly individuals [63]; elderly individuals with positive views of aging will adopt positive coping styles.

### Theoretical contribution

This research confirms the important roles of product usage perception, perceived ageing, and health promotion in predicting the intention of elderly individuals to continue using wearable devices. Previous studies have focused on the acceptance or adoption of wearable technology by elderly individuals, but there has been little research on subsequent, continuous use. Thus, this article fills a gap in the research on the willingness of elderly individuals to continue using wearable devices in elderly care institutions.

This article was based on the TAM and explored the factors that affect the willingness to continue using wearable devices from the perspective of product usage perception and individual characteristics. This proves that the TAM can also be applied to the willingness of elderly individuals to continue using wearable devices. Regarding the impact of perceived ease of use on continued use intention, the conclusions based on PLS-SEM and FsQCA results are inconsistent, indicating the complexity of perceived ease of use and providing insight for future research on technology perception.

This study proved the usefulness of combining SEM with FsQCA. SEM can indicate the strength of relationships between variables in the model, while FsQCA can indicate the configuration relationships between antecedent variables that produce specific results. These two complementary methods can be used to provide a basis for subsequent studies on the willingness to continue using wearable devices.

This article proves that perceived reliability does not affect health promotion, which is a new research finding. Past research on this relationship did not include samples of elderly individuals. Many factors can affect the health promotion of elderly individuals. This article provides new insight for research on the health promotion of elderly individuals.

Recently, some scholars have proposed that characteristics of ageing are important factors of individual behavioural intentions, but there are few studies on perceived ageing. The results of this article prove that it is an important factor for continued use intention, which motivates follow-up research.

### Practical importance

With the increasing popularity of wearable devices, applications for elderly care services have been proposed. We expect that their use will benefit pension institutions, senior citizens, and manufacturers. The PLS-SEM results indicated that the perceived usefulness and reliability of products affect the willingness to continue using wearable devices. Elderly care institutions can promote the functions and advantages of wearable technology to elderly individuals, regularly maintain the equipment and ensure safety, and emphasize the usefulness and effectiveness of continuous use of the technology. Reliability enables elderly individuals to achieve a clear understanding of wearable devices and will motivate elderly individuals to continue using them. In the long term, this use will improve the work efficiency of nursing staff, reduce nursing pressure, and enhance the competitiveness of enterprises.

In PLS-SEM, individual characteristics (perceived ageing and health promotion) were identified as important factors for the willingness to continue using wearable devices. The deterioration of body functions threatens the health of individuals. Continuous use of wearable devices can evaluate the health of elderly individuals and provide personalized data so that elderly individuals can change their health behaviours based on the data. Technical support can improve the daily life and independence of elderly individuals.

Compared with the PLS-SEM results, our FsQCA results provided new insights into the complex relationship between perceived ease of use and continued use intention. These solutions lead to a high willingness to continue using wearable devices among elderly individuals. This willingness has important implications for elderly care institutions and device manufacturers. For example, the research in this article shows that even if individuals have low levels of perceived aging, favourable product-use perceptions (ease of use, usefulness, and reliability) can increase health promotion, leading them to have a high willingness to continue using the devices (Path 5). Thus, wearable device manufacturers should develop user interfaces that are easy to understand and operate to ensure the ease of use of the devices. They should optimize the functions of the product to meet the preferences and health needs of elderly individuals. Additionally, they should improve the security and accuracy of the technology and guarantee product safety and privacy. Elderly care institutions should likewise provide technical guidance and education and training programs to elderly individuals so that they view the product and equipment as easy to use.

### Limitations and future research

This study has several limitations and suggests several future directions. First, our study used self-report data, and participants’ responses may be biased, such as with social desirability bias. Additionally, the cross-sectional design may lead to conclusions that do not accurately reflect the causal relationships among variables. Therefore, future studies need to use more accurate and reliable data collection methods to verify the reliability of the study findings.

Second, we analysed the influence of certain characteristics of elderly individuals in elderly care institutions on their willingness to continue using wearable devices. The participants in this study were elderly people in elderly care institutions in Anhui Province. Participation of elderly people from different regions would increase the diversity of the sample. Therefore, in the future, we need to collect data from a wider group of elderly people to enhance the generalizability and validity of our results.

Finally, we used PLS-SEM and FsQCA to study the relationship between symmetry and asymmetry and discussed the complex relationship between antecedent conditions and the willingness to continue using wearable devices. Few related studies have used these two methods to study the willingness to continue using wearable devices. However, in the future, FsQCA can be combined with other methods, such as multiple regression and grounded theory.

## Conclusion

The results of this article are highly important to researchers who study the willingness to continue using wearable devices among elderly individuals. We used SEM and FsQCA to further analyse how perceived ease of use, perceived usefulness, perceived reliability, perceived ageing, and health promotion influenced the willingness of elderly individuals to continue using wearable devices. FsQCA showed that the impact of perceived ease of use on the willingness to continue using wearable devices is complex, and perceived ageing was the core condition for the willingness to continue using wearable devices. The PLS-SEM and FsQCA methods used in this study enabled preliminary exploration of the willingness to continue using wearable devices among elderly individuals. In the future, combining these approaches with other research methods will provide a deeper understanding of the needs and views of elderly people who continue to use wearable devices.

## Data Availability

The authors confirm that the datasets used and/or analysed during the current study are available from the corresponding author on reasonable request.
